# Cationic Antimicrobial Peptides Promote Microbial Mutagenesis and Pathoadaptation in Chronic Infections

**DOI:** 10.1371/journal.ppat.1004083

**Published:** 2014-04-24

**Authors:** Dominique H. Limoli, Andrea B. Rockel, Kurtis M. Host, Anuvrat Jha, Benjamin T. Kopp, Thomas Hollis, Daniel J. Wozniak

**Affiliations:** 1 Department of Microbial Infection and Immunity, The Ohio State University, Columbus, Ohio, United States of America; 2 Department of Natural Sciences, Mars Hill University, Mars Hill, North Carolina, United States of America; 3 Medicine Administration, University of North Carolina Chapel Hill, Chapel Hill, North Carolina, United States of America; 4 Department of Pediatrics, The Ohio State University, Columbus, Ohio, United States of America; 5 Department of Biochemistry and Center for Structural Biology, Wake Forest University School of Medicine, Winston-Salem, North Carolina, United States of America; Massachusetts General Hospital, Harvard Medical School, United States of America

## Abstract

Acquisition of adaptive mutations is essential for microbial persistence during chronic infections. This is particularly evident during chronic *Pseudomonas aeruginosa* lung infections in cystic fibrosis (CF) patients. Thus far, mutagenesis has been attributed to the generation of reactive species by polymorphonucleocytes (PMN) and antibiotic treatment. However, our current studies of mutagenesis leading to *P. aeruginosa* mucoid conversion have revealed a potential new mutagen. Our findings confirmed the current view that reactive oxygen species can promote mucoidy *in vitro*, but revealed PMNs are proficient at inducing mucoid conversion in the absence of an oxidative burst. This led to the discovery that cationic antimicrobial peptides can be mutagenic and promote mucoidy. Of specific interest was the human cathelicidin LL-37, canonically known to disrupt bacterial membranes leading to cell death. An alternative role was revealed at sub-inhibitory concentrations, where LL-37 was found to induce mutations within the *mucA* gene encoding a negative regulator of mucoidy and to promote rifampin resistance in both *P. aeruginosa* and *Escherichia coli*. The mechanism of mutagenesis was found to be dependent upon sub-inhibitory concentrations of LL-37 entering the bacterial cytosol and binding to DNA. LL-37/DNA interactions then promote translesion DNA synthesis by the polymerase DinB, whose error-prone replication potentiates the mutations. A model of LL-37 bound to DNA was generated, which reveals amino termini α-helices of dimerized LL-37 bind the major groove of DNA, with numerous DNA contacts made by LL-37 basic residues. This demonstrates a mutagenic role for antimicrobials previously thought to be insusceptible to resistance by mutation, highlighting a need to further investigate their role in evolution and pathoadaptation in chronic infections.

## Author Summary

Antimicrobial peptides (AMPs) are produced by the mammalian immune system to fight invading pathogens. The best understood function of AMPs is to interact with the membranes of microbes, thereby disrupting and killing cells. However, the amount of AMP available during chronic bacterial infections may not be sufficient to kill pathogens (sub-inhibitory). In this study, we found that at sub-inhibitory levels, AMPs promote mutations in bacterial DNA, a function not previously attributed to them. In particular, we found that in the bacteria *Pseudomonas aeruginosa*, one AMP called LL-37 can promote mutations, which enable the bacteria to overproduce a protective sugar coating, a process called mucoid conversion. *P. aeruginosa* mucoid conversion is a major risk factor for those suffering from cystic fibrosis (CF), the most common lethal, heritable disease in the US. We found that LL-37 is able to produce these mutations by penetrating the bacterial cell and binding to the bacterial DNA. DNA binding disrupts normal DNA replication and allows mutations to occur. Furthermore, we observed LL-37 induced mutagenesis in processes apart from mucoid conversion, in both *P. aeruginosa* and *E. coli*. This suggests that AMP-induced mutagenesis may be important for a broad range of chronic diseases and pathogens.

## Introduction

Cystic fibrosis (CF) is the most common lethal, heritable disease in the US and results from mutations in the gene encoding the CF transmembrane conductance regulator. One of the most concerning effects of this mutation is altered anion transport of the airway epithelial cells resulting in increased susceptibility to infections and enhanced innate immune responses (reviewed in ref. [1]). The combinatorial effect of defects in the CF airway and chronic bacterial infections results in a hyper inflammatory environment dominated by an influx of polymorphonucleocytes (PMNs). Chronic pulmonary infections with *Pseudomonas aeruginosa* are a leading cause of death in CF patients [2], [3]. During the course of infection, *P. aeruginosa* often undergoes a phenotypic change from a non-mucoid to a mucoid appearance, which directly correlates with a worsening clinical prognosis [2], [4]. Mucoid conversion is characterized by the overproduction of the polysaccharide alginate, which confers a selective advantage for *P. aeruginosa* in the CF lung by providing recalcitrance to currently available therapeutics and host antimicrobials (reviewed in refs. [5], [6]).

Mucoid conversion occurs when the negative regulator of alginate synthesis, MucA, is genetically or physiologically disrupted [7], [8]. Up to 84% of mucoid isolates from patients possess mutations within *mucA* resulting in constitutive overexpression of alginate [9]–[12]. Hyper inflammation in the CF lung environment generates an abundance of mutagenic factors, which may be responsible for directly inducing *mucA* mutations. For example, PMNs and hydrogen peroxide (H_2_O_2_) elevate mucoid conversion *in vitro* by promoting mutagenesis [13]–[15]. However, the robust adaptive nature of the *P. aeruginosa* genome in chronic CF infections is evident and perpetuated by the appearance of mutator strains, which likely contribute to selection of mucoid variants (reviewed in [16]–[19]). Therefore, it will be of therapeutic utility to determine if specific host factors in CF promote *mucA* mutagenesis and investigate if intervention at this initial stage would prove effective.

This study aimed to examine the specific role of host inflammatory factors in promoting *mucA* mutations leading to mucoid conversion. PMNs derived from both healthy and CF individuals stimulated mucoid conversion independent of selection. Surprisingly, while reactive oxygen species (ROS) have the capacity to induce mucoid conversion *in vitro*, PMNs still efficiently promote mucoid conversion in the absence of an oxidative burst response. This led to the discovery that non-oxidative PMN components, including the antimicrobial peptide LL-37, are important for promoting mucoid conversion in CF. Importantly, LL-37 also elevated spontaneous rifampin resistance in *P. aeruginosa* and *E. coli*, indicating a new role for LL-37 as a bacterial mutagen. LL-37-induced mutagenesis required the translesion DNA polymerase, DinB (Pol IV); whose error-prone replication is responsible for generating *mucA* mutations. Using several independent methods, it was determined that, at sub-inhibitory concentrations, LL-37 enters *P. aeruginosa* cells, interacts with DNA, and promotes *mucA* mutations. Finally, conversion of *P. aeruginosa* to the mucoid phenotype then protects the bacterial cells from killing by lethal concentrations of LL-37. These data reveal a novel mechanism to describe how antimicrobial peptides interact with bacterial cells and demonstrate that LL-37 may promote mutations leading to persistence and chronic infection.

## Results and Discussion

### Development of a genetic scheme to measure the frequency of mucoid conversion and select for stable *P. aeruginosa* mucoid variants

The study of mucoid conversion in the laboratory has been hampered by the difficulty in isolating rare mucoid variants that arise in a population (∼1×10^−9^). To circumvent this problem, a system for selecting mucoid colonies was developed (Figure S1 and *Materials and Methods*). A promoterless gene encoding chloramphenicol-resistance (*cat*) was placed under control of the promoter of the alginate biosynthetic operon (*algD*) [20], [21] and integrated into the chromosome of non-mucoid, chloramphenicol sensitive *P. aeruginosa* strain PAO1 to generate PAO1*algD-cat* (WFPA934). Under this system, mucoid bacteria that are producing alginate and therefore transcribing *algD* will be chloramphenicol-resistant, allowing for selection of mucoid variants by growth on chloramphenicol-containing media (Figure S1A). By comparing the numbers of colonies on non-selective versus chloramphenicol media the relative frequency of mucoid conversion was determined.

To investigate the utility of this selection strategy, non-mucoid *P. aeruginosa* were exposed to a sub-inhibitory concentration (1/10 the minimum inhibitory concentration (MIC; 0.1 µM)) of H_2_O_2_ for one hour, followed by overnight recovery in media alone. Cultures are then serially diluted and plated on non-selective media to determine the total number of bacteria (∼1×10^10^) and whole culture plated on chloramphenicol containing media to determine the number of mucoid colonies (0–300 depending on treatment). The mucoid conversion frequency is then determined by dividing the number of mucoid colonies by the total. There was no significant difference among treatments in the total number of bacteria after the one-hour treatment or after the overnight recovery period, therefore changes in mucoid conversion frequencies are a direct result of induction of mucoid variants. In agreement with previous studies [13], [14], [22], treatment with H_2_O_2_ significantly increased the frequency of mucoid conversion (Figure S1B). While non-mucoid chloramphenicol isolates were observed, the frequency was very low (∼1.9×10^−10^) and was not altered by the addition of H_2_O_2_ (or any of the other mutagens used in this study, see below). Since the translesion DNA polymerase DinB (Pol IV) and defects in the mismatch repair protein MutS contribute to mucoid conversion [14], [15], [22], the role of these proteins was also examined. As predicted, *P. aeruginosa* isolates lacking *mutS* exhibited increased mucoid conversion frequencies and *dinB–* deficient isolates had severely impaired mucoid conversion (Figure S1B). Importantly, no reduction in growth was observed during the short treatment periods utilized and resulting mucoid variants were stable upon multiple passages, where approximately 80% possessed mutations in *mucA* (Table S1). Therefore, we were able to identify mucoid variants, which acquire mutations by direct induction versus selection based on resistance. This selection strategy now provides a unique opportunity to interrogate the CF factors that directly promote mutations leading to mucoid conversion.

### PMNs promote mucoid conversion independent of phagocytosis and the oxidative burst response

**Figure 1:**
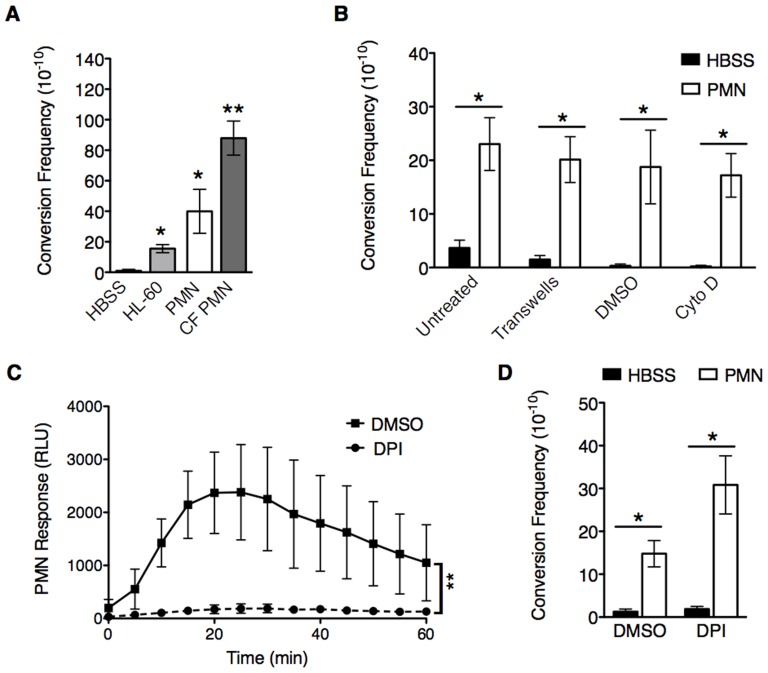
PMNs induce mucoid conversion independent of bacterial uptake and the oxidative burst response. **A,** opsonized PAO1*algD-cat* was incubated with either Hank's Buffered Saline (HBSS), HL-60 cells, or PMNs isolated from healthy human donors or CF patients followed by determination of the mucoid conversion frequency as described in Figure S1. **B,** phagocytosis of healthy PMNs was blocked by either physical separation from bacteria with transwells or treatment with cytochalasin D. In **C and D,** the PMN oxidative burst response was blocked by pretreatment of healthy PMNs with diphenyleneiodonium (DPI) (or DMSO, vehicle control) prior to incubation with PAO1*algD-cat*. The kinetic oxidative burst response of PMNs was measured by luminol (relative luminescent units (RLU)) in **C**, and the mucoid conversion frequency determined in **B** and **D**. All experiments were performed in triplicate on four to five independent occasions. Values are mean +/− standard error of the mean (SEM). For the statistical analysis of the kinetic oxidative burst response (**C**), the area under each curve was calculated ((RLU*min)^2^) for each treatment. Statistical analysis was carried out using an unpaired two-tailed student's *t*-test in **B** and **C** and Mann-Whitney test in **A** and **D**. (* p≤0.05, **p≤0.001).

To examine the role of PMNs in mucoid conversion, opsonized PAO1*algD-cat* was incubated in the presence of PMNs derived from three sources: peripheral PMNs from healthy or CF human donors, or an inducible promyelocytic cell line (HL-60) as described in *Supporting Material and Methods* (Text S1). Treatment with each cell type significantly increased the frequency of mucoid conversion (Figure 1A). A trend was also observed for increased mucoid conversion in CF PMNs compared to healthy PMNs from multiple donors; however, this was not a statistically significant difference. Moreover, *mucA* from mucoid isolates treated with healthy human PMNs harbored mutations (Table S1), demonstrating that human PMNs can induce mutations promoting mucoid conversion.

To investigate the mechanism(s) by which PMNs induce mucoid conversion, we first sought to determine if bacterial uptake is necessary. Phagocytosis was blocked by the addition of cytochalasin D (Figure 1B; confirmed by microscopy (Figure S2A)), or by separating PMNs from the bacteria with transwells (Figure 1B). Efficient mucoid conversion was maintained when phagocytosis was inhibited by either method, demonstrating the factors promoting mucoid conversion are released from the PMN. Since we hypothesize ROS generation is responsible for PMN-induced mucoid conversion, we sought to determine if inhibition of bacterial uptake affected the oxidative burst response. Surprisingly, both cytochalasin D and separation by transwells blocked the PMN oxidative burst (Figure S2B, C respectively), suggesting PMNs can promote mucoid conversion in the absence of ROS. To confirm this, PMNs were specifically inhibited by pretreatment with the NADPH oxidase inhibitor diphenyleneiodonium (DPI). While treatment of PMNs with DPI significantly inhibited ROS production (Figure 1C), this did not lead to inhibition of mucoid conversion (Figure 1D). Inhibition of oxidative burst was further confirmed with lucigenin and scopoletin, which measure O_2_• generation, and H_2_O_2_, respectively (data not shown). Collectively, these data demonstrate that PMNs deficient in generating ROS can still efficiently induce mucoid conversion.

### Non-oxidative PMN pathways promote mucoid conversion

**Figure 2:**
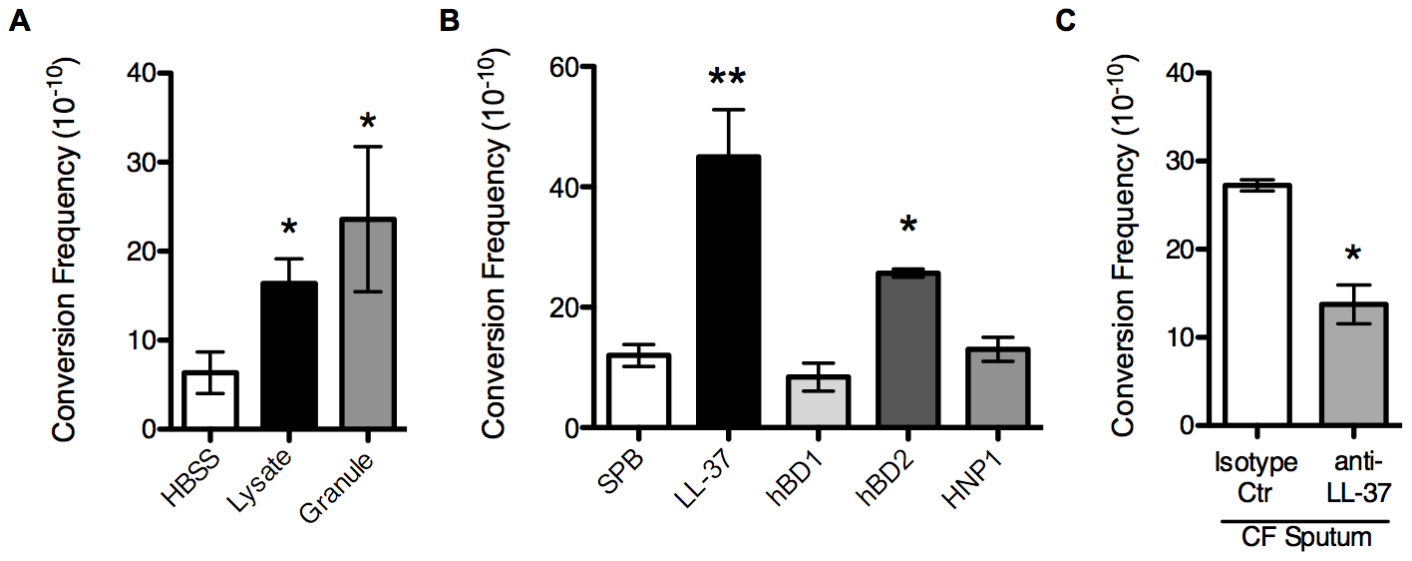
Non-oxidative PMN pathways promote mucoid conversion. The mucoid conversion frequency was determined upon treatment of PAO1*algD-cat* with PMN lysates or granule preparations (**A**), sub-inhibitory concentrations (0.25 µM) of LL-37, human beta defensin 1 and 2 (hBD1/2) and human neutrophil peptide 1 (HNP1) (**B**), or sputum isolated from CF patients (**C**). In **C**, sputum was immune-depleted with a monoclonal LL-37 antibody or mouse IgG_1_ isotype control antibody. All experiments were performed in triplicate on four independent occasions. Values are mean +/− SEM. Statistical analysis was carried out comparing HBSS or 10 mM sodium phosphate buffer (pH 6.2) (SPB) to PMN component treated in **A** and **B** and anti-LL-37 to isotype control treated sputum in **C**, using an unpaired two-tailed Mann-Whitney test (* p≤0.05, **p≤0.001).

The above data reveal that ROS-independent PMN factors may be important determinants of mucoid conversion. Moreover, while ROS clearly promote mucoid conversion *in vitro* and likely contribute to pathoadaptation *in vivo*, oxygen independent mechanisms may predominate in CF due to the hypoxic nature of the mucopurulent masses in the lumen of patient airways, where *P. aeruginosa* microcolonies reside [23], [24]. To test whether non-oxidative components are involved in mucoid conversion, PMN lysates that retained granular components but lack ROS were prepared (see Text S1. *Supporting Materials and Methods*). Treatment of PAO1*algD-cat* with PMN lysates or soluble components specifically isolated from granules increased the frequency of mucoid conversion compared to vehicle treated *P. aeruginosa* (Figure 2A). Moreover, PMN lysates and granule fractions promoted mutations within *mucA*, indicating non-oxidative PMN components possess mutagenic capacity (Table S1). To investigate which granule component(s) are responsible for mucoid conversion, PAO1*algD-cat* was incubated with sub-inhibitory concentrations (0.25 µM) of purified AMP including the human cathelicidin, LL-37, beta defensins (hBD) 1 and 2, and human neutrophil peptide 1 (HNP1). LL-37 and hBD2 treatment increased the frequency of mucoid conversion approximately four-fold and two-fold respectively compared to buffer controls, while hBD1 and HNP1 did not exhibit any effect (Figure 2B). Together these data reveal a novel property for non-oxidative PMN granular components, including specific antimicrobial peptides, in promoting *P. aeruginosa* mucoid conversion.

LL-37 is a human cationic host defense molecule elevated in CF sputum and found in the granules of PMNs and at mucosal surfaces [25]. In addition to possessing a broad range of antimicrobial action against bacteria, viruses, and fungi, LL-37 possess immunomodulatory and anti-biofilm activities at sub-inhibitory concentrations [26]–[28]. In PMNs, the propeptide form of LL-37 is stored within the specific granules and upon stimulation is released to the extracellular environment and cleaved to the mature form by serine proteases [29]. Since the PMN factors primarily responsible for mucoid conversion are released from the PMN into the extracellular environment (Figure 1B) and we were able to confirm the presence of mature LL-37 in PMN lysate preparations by immunoblot analysis (Figure S3), we hypothesized that LL-37 may promote mucoid conversion in CF and focused current studies on LL-37.

### LL-37 contributes to *P. aeruginosa* mucoid conversion in CF

Despite the presence of elevated LL-37 in CF sputum, it has been argued that in the CF pulmonary environment some AMPs may not function optimally due to high salt concentrations [30], [31] or may be sequestered by extracellular DNA and F-actin bundles [32]. Therefore, to determine if LL-37 mediated mucoid conversion is a process capable of functioning in the CF pulmonary environment, the impact of sputum isolated from CF patients on mucoid conversion was investigated. Sputum isolated from four different CF individuals induced an average mucoid conversion frequency of 3×10^−9^ (Figure 2C). To determine if LL-37 contributes to sputum induced mucoid conversion, LL-37 was immune-depleted from each sputum sample. The mucoid conversion frequency decreased significantly upon depletion with antibodies directed toward LL-37, compared to an isotype control antibody (Figure 2C), demonstrating LL-37 contributes to mucoid conversion in CF sputum. However, immune-depletion of LL-37 did not completely abolish mucoid conversion. This is likely a combination of incomplete elimination of LL-37 from CF sputum, as well as potential contribution of other inflammatory factors in sputum to mucoid conversion (oxidative and nitrosative stress, other AMP, etc). Moreover, after incubation of *P. aeruginosa* with CF sputum, no change in the overall viability of the bacteria was observed (data not shown). These data suggest that antimicrobial factors present in CF sputum may not be present at bioavailable levels sufficient to control *P. aeruginosa* infections, but are sufficient to promote conversion to the mucoid phenotype.

### LL-37 induces bacterial mutagenesis

**Figure 3:**
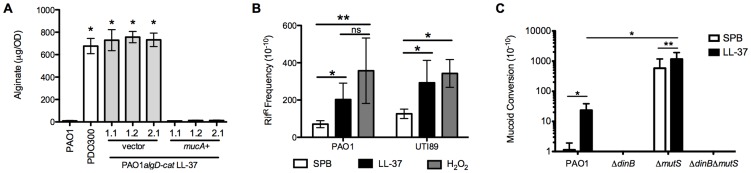
LL-37 induces bacterial mutagenesis in a DinB-dependent manner. **A**, *mucA* was expressed in PAO1*algD-cat* LL-37 1.1, 1.2 and, 2.1 on an arabinose-inducible plasmid (pHERD). Alginate was isolated from strains containing either empty pHERD (vector) or pHERD*mucA* (*mucA^+^*), grown on 0.5% arabinose, and the amount of alginate produced measured by a carbazole assay and compared to the parental non-mucoid PAO1*algD-cat* and mucoid PDO300. **B**, The frequency of rifampin resistance (Rif^R^) of non-mucoid PAO1*algD-cat* (*P. aeruginosa*) and UTI89 (*E. coli*) following treatment with sub-inhibitory doses of LL-37 or H_2_O_2_ was determined. **C**, Non-mucoid PAO1*algD-cat*, PAO1*algD-catΔdinB*, PAO1*algD-catΔmutS* and PAO1*algD-catΔmutSΔdinB* were treated with sub-inhibitory LL-37 or SPB and the mucoid conversion frequency determined. Experiments were performed in triplicate on four independent occasions. Values are mean +/− SEM. Statistical analysis was carried out using an unpaired two-tailed student's *t*-test in **A** and **C** and Mann-Whitney test in **B**. (* p≤0.05, **p≤0.001).

To investigate how LL-37 promotes mucoid conversion, we first interrogated the primary mechanism of mucoid conversion observed in CF *P. aeruginosa* isolates, mutagenesis of the anti-sigma factor encoding gene *mucA*. We subjected ten LL-37-derived mucoid isolates to *mucA* sequence analysis (Table S2). All isolates possessed mutations within *mucA* and 70% of these were frameshifts predicted to eliminate *mucA* function. Mucoid clinical isolates from CF patients possess a range of *mucA* alterations; however, frameshift mutations and C to T transitions are the most common [33]–[36], a pattern represented here in LL-37 treated mucoid isolates (Table S2). To confirm the alterations in *mucA* were responsible for the mucoid phenotype, *mucA* was expressed on an arabinose inducible plasmid (pHERD20T*mucA*) in representative isolates with unique *mucA* alleles (WFPA934 LL-37 1.1 (ΔC at 184), 1.2 (C→A at 531), and (ΔT at 470)). The phenotype of each isolate harboring pHERD20T*mucA* was complemented to the non-mucoid phenotype upon growth on arabinose (Figure 3A), indicating these mutations are directly responsible for LL-37-dependent mucoid conversion.

To determine if LL-37 promotes global bacterial mutagenesis, the frequency of LL-37-induced rifampin resistance (Rif^R^) was examined. Upon treatment of non-mucoid PAO1 with sub-inhibitory concentrations of LL-37 a moderate, but significant increase in Rif^R^ was observed compared to cells treated with buffer. The frequency of acquisition of Rif^R^ was similar to control treatments with H_2_O_2_ (Figure 3B), which is known to promote global mutagenesis in a range of microorganisms [37]. Furthermore, LL-37 also elevated the acquisition of Rif^R^ in *E. coli*, demonstrating this mechanism of mutagenesis is not specific to *P. aeruginosa* (Figure 3B). Collectively, these data demonstrate for the first time that LL-37 can function as bacterial mutagen and may contribute to the generation of mutations during chronic infection.

### The translesion DNA polymerase DinB is essential for LL-37 induced mucoid conversion

To uncover the mechanism by which LL-37 induces mutations, we investigated the DNA repair enzymes MutS and DinB, due to their previously identified role in both *P. aeruginosa* and *E. coli* mutagenesis [14], [15], [22], [38]. The mucoid conversion frequency of Δ*mutS*, Δ*dinB* and double Δ*mutS*Δ*dinB* mutants upon treatment with LL-37 was determined (Figure 3C). In the *mutS* mutant the spontaneous mutation frequency was increased nearly 1000-fold and this was further elevated with LL-37 treatment. Of notable significance, mucoid conversion was almost eliminated in a *dinB*-deficient strain, independent of the *mutS* status. These results illustrate an important role for DNA repair proteins in LL-37-induced mucoid conversion.

Cell envelope stress has been linked to production of intracellular ROS, which induce mutagenesis and bacterial SOS response genes [39]. Since DinB is a member of the SOS regulon [15]; we initially hypothesized that LL-37 interactions with the *P. aeruginosa* cell envelope would result in membrane stress, leading to induction of SOS response genes, including *dinB*. Therefore, the expression of *P. aeruginosa* regulators of membrane and SOS responses, *algT/U* (σ^E/22^), and *lexA*, respectively, were evaluated by quantitative real time PCR (qRT-PCR) following LL-37 exposure. However, treatment of non-mucoid PAO1 with sub-inhibitory concentrations of LL-37 did not significantly alter the expression of *lexA* or *algT/U*, whereas positive control treatments did (mitomycin C and D-cycloserine, respectively) (Table S3). Surprisingly, expression of *dinB* was also not altered by LL-37 treatment (Table S3). Combined with the observation that DinB is required for spontaneous generation of *mucA* mutations (buffer treated, Figure 3C), these results suggest that basal levels of DinB present during normal replication are sufficient to promote mutagenesis leading to mucoid conversion. We propose that LL-37 elevates mutagenesis in *P. aeruginosa* by a mechanism independent of membrane or SOS stress responses.

### Sub-inhibitory concentrations of LL-37 enter the *P. aeruginosa* cytosol and interact with DNA

**Figure 4:**
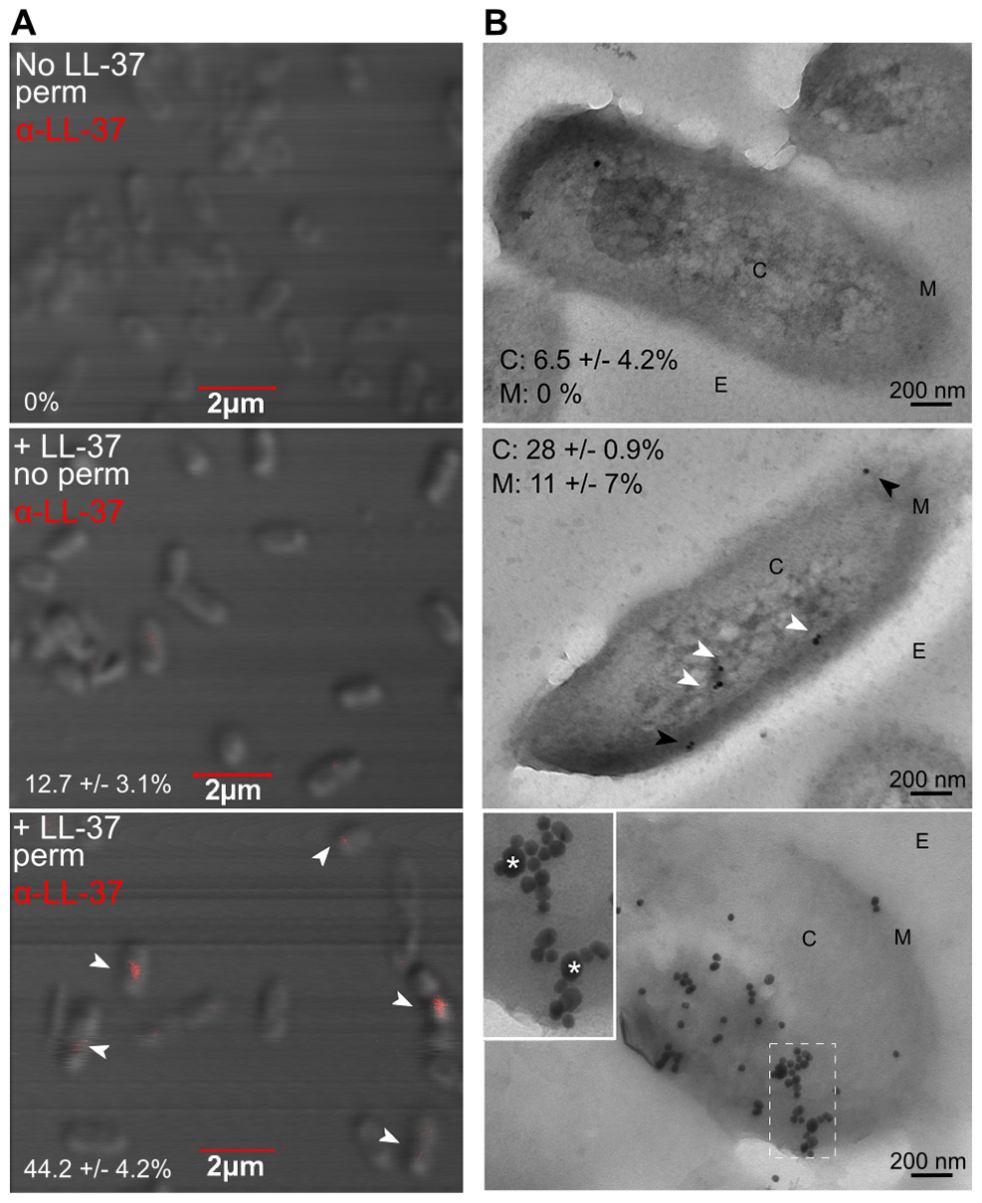
Sub-inhibitory concentrations of LL-37 enter the bacterial cytosol and interact with DNA. Visualization of LL-37 localization in *P. aeruginosa* cells using confocal (**A**) and transmission electron microscopy (TEM, **B**). Bacteria were treated +/− sub-inhibitory LL-37, fixed and in **A**, permeabilized (top and bottom panels), stained for LL-37 (AlexaFluor647, red) (indicated by white arrows) and visualized by 100× objective. The percentage of cells counted on triplicate coverslips with LL-37 labeling inside the cell is indicated. In **B,** bacteria were treated with sub-inhibitory LL-37 (middle and bottom panels) or untreated (top) and cells were labeled with anti-LL-37 antibody conjugated to Protein G colloidal gold (20 nm). In the bottom panel, cells were additionally labeled with anti-dsDNA antibody conjugated to Protein G colloidal gold (10 nm). C: cytosol, M: membrane and E: extracellular. White arrows: cytosolic LL-37, Black arrows: membrane LL-37. In the bottom panel, boxed images are 2× magnified and LL-37 labeling is indicated by *. Twenty random, blind images were taken for each condition and the percentage of LL-37 labeling in the membrane and in the cytosol is indicated.

Bacterial DNA has been proposed to be an alternative target for a subset of AMPs, whereby peptides gain entry to the cytosol and bind bacterial DNA, resulting in subsequent disruption of DNA or protein synthesis [40]–[45]. Since membrane stress and SOS response pathways were not induced by LL-37, we explored an alternative hypothesis that LL-37 may induce translesion DNA synthesis and mutagenesis by interacting directly with genomic DNA. The structure of LL-37 resembles classical cell-penetrating peptides and it has been postulated that LL-37 may enter both prokaryotic and eukaryotic cells. Lande *et al* demonstrated that LL-37 is capable of trafficking into dendritic cells, providing evidence that LL-37 can penetrate eukaryotic cells [46]. While multiple reviews have generalized this observation to include all cell types, it has yet to be formally determined if LL-37 can enter the cytosol of bacterial cells [27], [45]. Therefore, the capacity of sub-inhibitory concentrations of LL-37 to enter the *P. aeruginosa* cytosol was examined utilizing fluorescent confocal- and transmission-electron microscopy (TEM). Non-mucoid PAO1 was treated with LL-37, fixed, and labeled with anti-LL37. For confocal microscopy, the localization of anti-LL37 labeling of permeabilized PAO1 (to allow antibody access to the cytosol) was compared to non-permeabilized cells. Non-permeabilized cells showed a weak, diffuse signal on the surface, compared to permeabilized cells, which demonstrated a stronger, punctate signal in the center of the cell (indicated by white arrows, Figure 4A). This was clearly visible only in the cytosol, when sequential images where taken along the extent of the Z plane of the cells (shown in Movie S1). With transmission electron microscopy (TEM), LL-37 labeling was visible in the cytosol of approximately 28% (white arrows) of cells and in the membrane of 11% (black arrows)(Figure 4B, center).

These data reveal for the first time that LL-37 can gain entry to the cytosol of *P. aeruginosa*. However, intracellular LL-37 was visualized in only a subset of cells. Of note, 43% of cells with cytosolic LL-37 labeling, as visualized by confocal microscopy, appeared to be actively dividing (Figure 4A). At lethal concentrations, LL-37 readily interacts with the septum of diving cells [47]; therefore active replication may also play a role in LL-37 gaining access to the *P. aeruginosa* cytosol at sub-inhibitory concentrations. Several models have been proposed to explain how AMPs interact with bacterial membranes, but accumulating evidence for LL-37 supports the Shai-Matsuzaki-Huang model [28], [48]–[51]. In this model, peptides carpet the outer leaflet of the bacterium and integrate into the membrane. This is followed by a transient pore forming stage, where lipids and peptides are transported to the inner leaflet, resulting in collapse of membrane fragments and disruption of the membrane. In some cases, transient pore formation results in diffusion of peptides into the cytosol, where peptides can then interact with intracellular targets [48]. At sub-inhibitory concentrations, transient pore formation may occur without significant disruption of the membrane or loss of cell viability.

To determine if bacterial DNA might be an intracellular target of LL-37, TEM experiments were performed as described previously, with a second label added for double-stranded DNA. 76% of intracellular LL-37 was localized with *P. aeruginosa* DNA (Figure 4B, bottom panel). Electrophoretic mobility shift assays (EMSA) were also performed, where LL-37 treatment resulted in a shift of all DNA tested with similar affinity (apparent K_D_ = 12.8 µM, Figure S4), further demonstrating that LL-37 binds non-specifically to *P. aeruginosa* DNA. It was observed that upon the addition of increasing amounts of LL-37 a threshold concentration was reached where no migration of DNA was observed, instead of a step-wise migration, a phenotype that has been observed for other short DNA binding peptides [41], [52].

**Figure 5:**
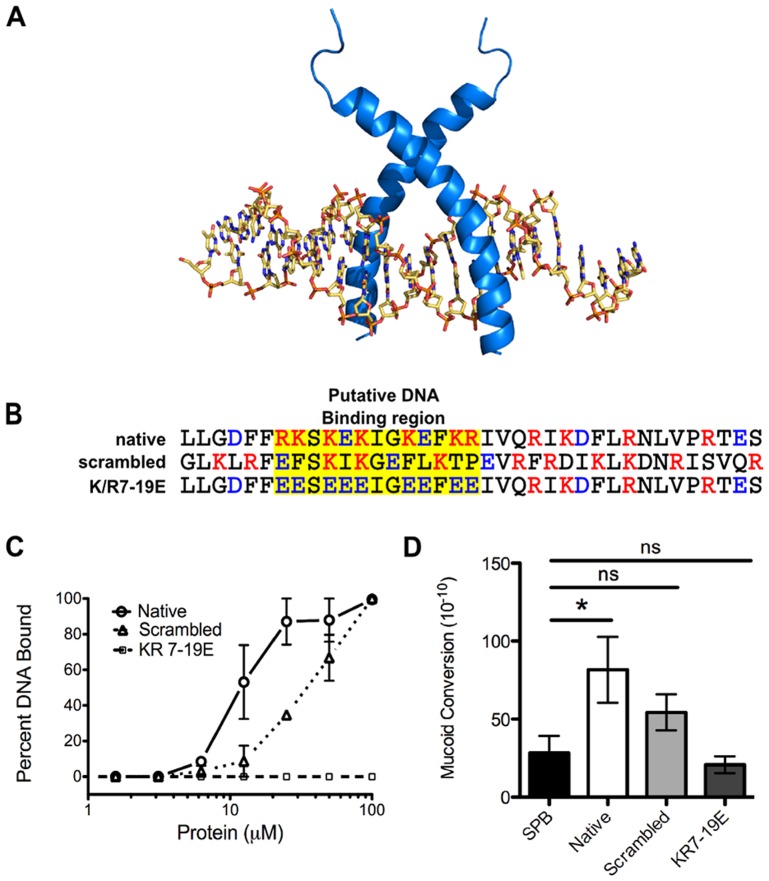
LL-37 DNA binding promotes mucoid conversion. **A,** homology modeling of LL-37 bound to *B*-DNA was performed manually on the basis of the backbone atomic coordinates of the homologous protein, sterol regulatory element binding protein, bound to DNA. In **B**, amino acid sequences of native LL-37 and synthetic derivatives are represented and the putative DNA binding region is indicated in yellow. Red: positive residues, blue: negative residues. In **C**, the percent of DNA bound by LL-37 derivatives was calculated from electrophoretic mobility shift assays (representative images in Figure S4), where densitometry was performed on each image using ImageJ. **D** represents the mucoid conversion frequency after treatment with LL-37 derivatives. Values are mean +/− SEM. Experiments were performed in triplicate on three independent occasions and statistical analysis was carried out using an unpaired two-tailed student's *t*-test (**C**) or Mann-Whitney test (**D**). (* p≤0.05).

To investigate how LL-37 may interact with DNA and whether DNA binding is necessary for mucoid conversion, a structure-based search of LL-37 revealed homology to eukaryotic transcription factors containing basic region leucine zipper (bZIP) motifs. A three-dimensional model of LL-37 bound to DNA suggested that the amino-terminal basic residues of LL-37 occupy positions suitable for interactions with the negatively charged phosphate groups of the DNA backbone (Figure 5A). To investigate LL-37/DNA interactions, two synthetic peptides were generated: an LL-37 variant with the amino acid sequence randomly scrambled and a variant with the basic residues in the predicted DNA binding region replaced with glutamate residues (K/R7-19E, Figure 5B). Scrambled LL-37 had a moderate loss of DNA binding, as well as a decrease in the ability to induce mucoid conversion (Figure 5C and D). When basic residues within the putative DNA binding region were modified, a complete loss of both DNA binding and mucoid conversion was observed (Figure 5C and D). These data suggest that LL-37/DNA interactions are required for LL-37 to promote mucoid conversion. Studies are currently underway to further define LL-37/DNA interactions, their impact upon DinB and/or the DNA replisome, and the precise mechanism of mutagenesis.

### LL-37 contributes to pathoadaption of *P. aeruginosa*

**Figure 6:**
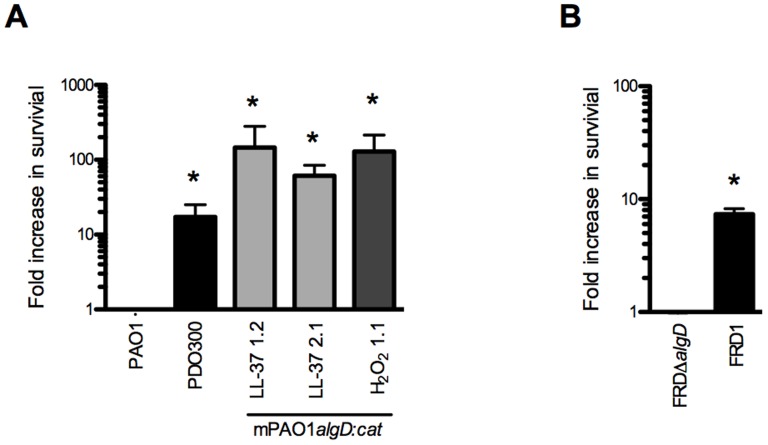
Alginate provides *P. aeruginosa* protection from lethal concentrations of LL-37. The survival of *P. aeruginosa* strains (non-mucoid parental PAO1*algD:cat* (PAO1), mucoid PDO300, mucoid strains derived from LL-37 (1.2 and 2.1) or H_2_O_2_ (1.1) treatment (**A**), FRDΔ*algD* (non-mucoid) and FRD1 (mucoid) (**B**)) was determined following treatment with lethal concentrations of LL-37 (6.25 µM) and is represented as a fold increase from the non-mucoid isogenic strain. Experiments were performed in triplicate on three or four independent occasions. The mean +/− SEM is indicated. Statistical analysis was carried out to compare the survival of mucoid isolates compared to the non-mucoid isogenic strain (PAO1 or FRDΔ*algD*), using an unpaired two-tailed student's *t*-test to compare in (* p≤0.05).

Herein, we show that LL-37 induces mucoidy but it is unknown if this conversion provides *P. aeruginosa* adaptive protection from lethal concentrations of LL-37. We observed a 10-fold increase in survival of two mucoid isolates derived from LL-37 treatment when compared with parental non-mucoid PAO1 (Figure 6A). To determine if protection was dependent upon prior treatment with LL-37, a H_2_O_2_-derived mucoid isolate, and a genetically engineered mucoid strain, PDO300 (PAO1Δ*mucA22*) were examined and both showed enhanced resistance to LL-37 mediated killing (Figure 6A). These data demonstrate that conversion to the mucoid phenotype provides *P. aeruginosa* protection from lethal concentrations of LL-37, independent of prior exposure to LL-37. Since *mucA* inactivation and subsequent *algT/U* induction controls an entire regulon, of which only a subset are dedicated to alginate production, it was necessary to determine if alginate overproduction is specifically responsible for LL-37 protection. FRD*mucA22*Δ*algD* (deficient in alginate production) was 10-fold more susceptible to LL-37 compared to the isogenic mucoid parental strain FRD1 (*mucA22*, Figure 6B). These data demonstrate that alginate overproduction contributes to protection of *P. aeruginosa* from LL-37 killing and this might promote *P. aeruginosa* pathoadaptation in the CF pulmonary environment.

**Figure 7:**
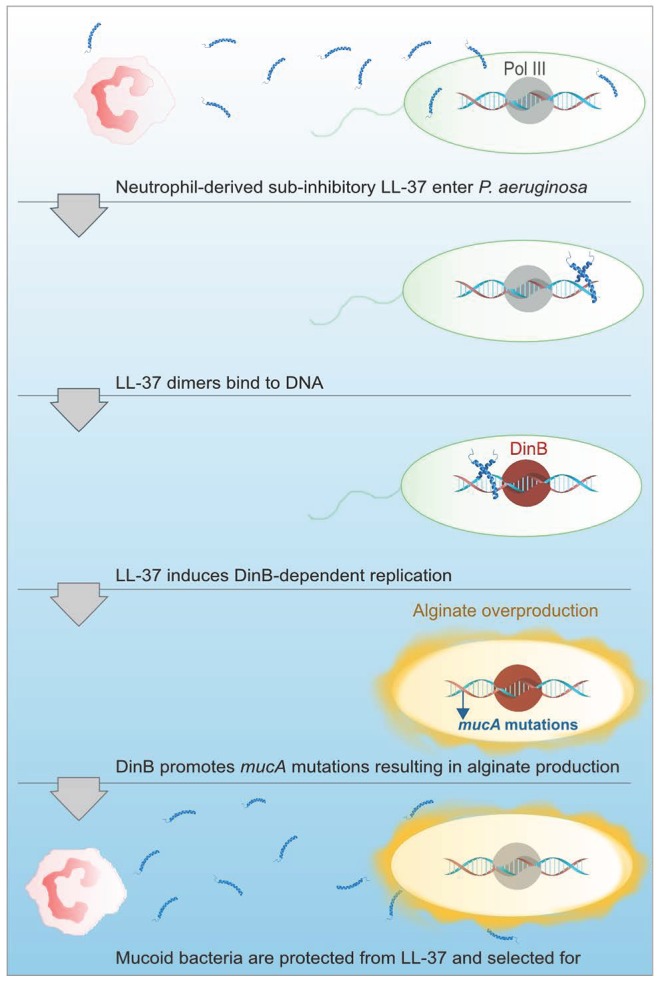
Proposed model of LL-37 induced mutagenesis. At sub-inhibitory concentrations, LL-37 can penetrate *P. aeruginosa* cells and enter the bacterial cytosol, where LL-37 dimers then bind to DNA. DNA binding by LL-37 then promotes DinB-dependent replication, which potentiates mutations in *mucA* leading to mucoid conversion. Alginate overproducing bacteria are then protected from lethal concentrations of LL-37 and mucoid variants are selected for and persist in CF.

Collectively, these data provide evidence for an additional role for LL-37 beyond the antimicrobial, anti-biofilm, and immunomodulatory functions previously described. We demonstrate that at sub-inhibitory levels, LL-37 promotes bacterial mutagenesis, which may contribute to evolution and pathoadaptation during chronic infections. Importantly, LL-37 induced mutations within *mucA* mimic what is observed in mucoid *P. aeruginosa* strains isolated from the CF airway and the sub-inhibitory conditions utilized may be representative of the level of bioavailable peptide present in the CF pulmonary environment. Furthermore, LL-37 induced mutagenesis of *mucA* leading to mucoid conversion was modulated exclusively by the error-prone polymerase DinB. We were intrigued by the stringent dependence upon DinB in this process, particularly since previous studies demonstrate DinB is not required for spontaneous or UV-induced Rif^R^ [15]. We observed that LL-37 can promote Rif^R^ in both *P. aeruginosa* and *E. coli*, suggesting perhaps the mechanism for acquisition of LL-37-induced *mucA* mutations and Rif^R^ occur via different pathways. Moreover, LL-37 did not increase the expression of DinB under these conditions demonstrating basal levels of DinB are sufficient to promote mutagenesis. Typical translesion DNA synthesis occurs when the replisome stalls upon encountering damaged DNA or a challenging template and low-fidelity polymerases like DinB will displace Pol III in order to perpetuate replication [53]. Since LL-37 interactions with DNA are required for LL-37-incuded mutagenesis we postulate that LL-37 presents a physical barrier that stalls Pol III, inducing a switch to DinB, whose error-prone replication promotes mutagenesis (see Figure 7 for model). EMSAs suggest that LL-37 non-specifically interacts with DNA; however, peptides have been identified which specifically interact with DNA repair intermediates, such as Holliday junctions [54], [55]. Therefore, an alternative hypothesis could be that LL-37 perturbs effective DNA repair by binding to repair intermediates. In this regard, Overhage *et al* performed microarray experiments with sub-inhibitory concentrations of LL-37 and identified changes in expression of genes involved in alginate regulation and DNA repair [26]. However, differences in the conditions utilized in these experiments and a lack of convergence upon a single pathway do not support any one hypothesis. Additionally, sub-inhibitory levels of LL-37 have been found to stimulate expression of the capsule synthesis operon in Group A Streptococci [56]. Together these studies suggest additional functions for sub-inhibitory levels of LL-37 impacting the expression of virulence genes. While the current study identified stable variants generated by mutagenesis, it is interesting to speculate how transient gene expression may impact their generation and selection in the host environment and further studies are clearly warranted.

While data presented here demonstrate PMNs can promote mucoid conversion in the absence of an oxidative burst response, it is likely that mucoid conversion in the CF lung results from a combination of non-oxidative, oxidative, and nitrosative stresses. Moreover, other chronic pneumonias, such as COPD, are also characterized by elevated levels of PMNs, generally on the order of two to three-fold higher than healthy controls [57], [58]. Mucoid *P. aeruginosa* have been isolated from patients with COPD, therefore, the mechanisms elucidated in this study may be important in other infections where chronic inflammation ensues [59]. Bronchial alveolar lavage fluid recovered from CF patients can have up to a 380-fold increase in PMNs recovered compared to healthy patients, even in patients without symptoms of an active infection [60]. Moreover, elevated levels of PMNs are detectable in CF newborns, which persist and undergo cycles of exacerbation throughout the life-time of the patient [61]. We therefore hypothesize that persistent exposure of *P. aeruginosa* to chronic inflammation for decades significantly contributes to microbial pathoadaptation in CF patients. Future investigation of how these inflammatory factors function in combination to promote *P. aeruginosa* pathoadaptation will be important for comprehensive understanding of these processes and the development of rational therapeutics for chronic infections.

Aggressive antibiotic and anti-inflammatory use over the past decade has drastically improved the life expectancy and disease outcome for CF patients. However, increased acquisition of antibiotic resistance mechanisms is presenting a significant challenge for future treatment options [62]. Many are turning towards cationic antimicrobial peptides as a promising alternative for developing antimicrobials, as they are thought to be relatively insusceptible to the development of resistance mechanisms by mutations [63]. This study demonstrates that some antimicrobial peptides may instead act to promote mutagenesis and the acquisition of resistance at sub-inhibitory levels. These data reinforce how important it is to consider the impact of current and novel treatments and the host immune response on evolution of microbial communities during chronic infections.

## Materials and Methods

### Ethics statement

Human PMNs and serum were obtained from healthy adult human donors according to the protocol approved by The Ohio State University Biomedical Sciences Institutional Review Board (2009H0314), where informed consent was obtained from all donors. Sputum, PMNs and serum were obtained from CF patients (adults and minors) according to the protocol approved by The Nationwide Children's Hospital Institutional Review Board (IRB12-00405) with informed consent obtained from all adult donors and from the parents/guardians of minors who participated. For children between the age of 9 and 18 an assent form was also obtained.

### Mucoid conversion studies

Overnight cultures of WFPA934 (PAO1*algD-cat*, Table S4) were diluted into fresh M63 media and grown to mid-log phase. 2×10^8^ colony forming units (CFU)/ml were resuspended in Hank's buffered saline (HBSS) or 10 mM sodium phosphate buffer (pH 6.2, SPB), as indicated and treated with sub-inhibitory concentrations of antimicrobials for one hour. Reactive oxygen species were used at 1/10 the minimum inhibitory concentration (MIC): hydrogen peroxide, 0.1 µM, hypochlorous acid, 0.1 µM, and paraquat to generate superoxide, 100 mM, antimicrobial peptides (LL-37, human beta-defensin 1, human beta-defensin 2 and human neutrophil peptide 1) were used at 0.25 µM (PeproTech, Rocky Hill, NY). Human PMNs and serum were isolated according to previously described protocols [64]. Mid-log phase *P. aeruginosa* suspensions were opsonized with 10% fresh human serum at 37°C for 30 min, added (MOI of 50) to the wells and centrifuged at 100 *g* for 2 min at 4°C to synchronize the infection. PMN treatments were performed according to previously described protocols and are described in detail in *Supporting Materials and Methods* (Text S1). *P. aeruginosa* cells were then washed twice and resuspended in M63 media for an overnight growth recovery period. Cultures were then serially diluted for plating on non-selective *Pseudomonas* isolation agar (PIA) and incubated at 37°C overnight to determine the total CFU and plated straight onto PIA containing chloramphenicol (250 µg/ml) and incubated at 37°C for 48 hours to determine the number of mucoid CFU. The mucoid conversion frequency was then determined by dividing the number of mucoid variants by the total number of CFU.

### CF sputum samples

CF sputum samples were collected by spontaneous expectoration from patients attending Nationwide Children's Hospital in Columbus, OH (IRB12-00405). The samples were diluted 1∶1 in buffer containing 140 mM NaCl, 10 mM Tris, 0.2 mM CaCl_2_ (pH 7.4) and physically disrupted by pipetting up and down with decreasing sized serological pipettes, followed by pushing through decreasing sized needles until sample is easily pushed through a 27 gauge needle. Samples were then centrifuged (10 min; 15,500 *g*) to pellet the remnant cells and bacteria. For immune-depletion of LL-37, sputum was incubated with 1.6 µg/ml monoclonal anti-LL37 antibody (Santa Cruz Biotechnology, Dallas, TX) or mouse IgG_1_ isotype control antibody (R & D Systems, Minneapolis, MN) overnight at 4°C. The antibody/antigen complex was then pulled-down with Protein A/G Agarose Beads (Thermo Scientific, Rockford, IL) according to the manufacturer's instructions and confirmed by immunoblot analysis (*See Text S1. Supporting Materials and Methods*).

### PCR and sequencing

Genomic DNA was harvested from the mucoid variants isolated in the mucoid conversion assay using the Wizard genomic purification kit (Promega). PCR amplification was performed using *mucA*-specific primers *mucA*upF and *mucA*dnR (Table S5). After verification of PCR product by agarose gel electrophoresis, The Ohio State University Medical Center Nucleic Acid Core sequenced the PCR products using Sanger sequencing techniques with mucAupF, mucAdnR, and mucA1F21. The sequence data produced for mucoid variants were then aligned with *mucA* gene sequence of the non-mucoid PAO1*algD-cat* parental to determine if and/or where mutations occurred in the *mucA* gene of the variants.

### Complementation analyses and alginate quantification

The *mucA* allele was cloned into the shuttle vector pHERD20T [65] for complementation with gene expression driven by the pBAD arabinose-inducible promoter. *P. aeruginosa* strains were grown at 37°C on PIA plates supplemented with carbenicillin and 0.1% (wt/vol) arabinose for 24 h. Bacterial growth was removed from plates with phosphate-buffered saline (PBS) and the optical density at 600 nm (OD600) of the bacterial suspension in PBS was measured. Alginate was isolated and measured by a standard carbazole assay as previously described [66], [67].

### Rifampin assays

Overnight cultures were diluted into fresh M63 media for *P. aeruginosa* or LB for *E. coli* and grown to mid-log phase. 2×10^8^ CFU/ml were resuspended in 10 mM sodium phosphate buffer (pH 6.2)(SPB), and treated with sub-inhibitory concentrations of LL-37 or H_2_O_2_ (1.25 µM or for *P. aeruginosa* and 6.25 µM for *E. coli.*). Cells were then washed twice and resuspended in media for an overnight growth recovery period. Cultures were then serially diluted for plating on non-selective PIA or LB and plated straight onto media containing 100 µg/ml rifampin (Rif) and incubated at 37°C for 24 hours. The Rif resistance frequency was then determined by dividing the number of Rif resistant variants by the total number of CFU.

### Microscopy studies

For determination of localization of sub-inhibitory concentration of LL-37 by confocal microcopy, PAO1*algD-cat* was grown to mid-log phase and 1×10^6^ cells were incubated with 0.25 µM LL-37 or SPB for one hour. Cells were washed, fixed in 4% paraformaldehyde for 10 min, permeabilized with 0.2% Triton-X for 1 min, blocked with 2% bovine serum albumin (BSA) and stained with anti-LL-37 antibody (Santa Cruz) (1∶100) conjugated directly to Alexa Fluor 647 (Invitrogen). Bacteria were wet-mounted onto coverslips and visualized by confocal microscopy (Olympus FV 1000 Spectral) using a 100× oil objective. For quantification, 100 cells were chosen at random and intracellular and membrane-associated peptides counted and averaged by two readers blinded to the treatment conditions. For transmission electron microscopy, PAO1 was grown to mid-log phase and 1×10^6^ cells were incubated with 0.25 µM LL-37 or SPB for one hour. Cells were washed and fixed in 4% paraformaldehyde for 12 h. Free aldehyde was quenched by the addition of 0.1 M glycine for 20 min and cells were resuspended in 0.2 M sucrose and incubated at 4°C overnight. Samples were embedded in LR white, cut into ultrathin sections (Leica EMU 6 ultramicrotome) (60–90 nm) and collected into formvar-coated nickel grids. Sections were stained by anti-LL37 (Santa Cruz) antibody conjugated to Protein-G colloidal gold (20 nm)(1∶500)(EY Laboratories) and/or anti-dsDNA (Abcam) conjugated to Protein-G colloidal gold (10 nm, 1∶100, EY Laboratories). Sections were viewed by transmission electron microscopy (FEI Tecnai G2 Spirit) operating at 80 kV. Twenty images were chosen at random and intracellular and membrane-associated peptides counted and averaged by two readers blinded to the treatment conditions.

### Structure analysis and molecular modeling

A structural based homology search was performed using the DALI server. Homology modeling of LL-37 bound to *B*-DNA was performed manually on the basis of the backbone atomic coordinates of the homologous protein, sterol regulatory element binding protein, bound to DNA, whose crystal structure is known (pdbid: 1am9) [68].

### LL-37 susceptibility assay

2×10^8^ CFU of mid-log phase *P. aeruginosa* strains were incubated with lethal doses of LL-37 (2.5 µM) of LL-37 for 1 hour at 37°C washed, serially diluted and plated on PIA to determine the total CFU.

### Statistical analysis

Results of mutagenesis studies presented significant variation (including spontaneous untreated controls) in accordance with previous observations [69]. Therefore all mutagenesis studies (including rifampin resistance) were performed in triplicate on at least four independent occasions. For all statistical analyses, data were tested for normality using Prism Version 5.0b. Data determined to be normally distributed were analyzed for statistical significance using parametric unpaired two-tailed students *t*-test in Prism. Data not normally distributed were analyzed using non-parametric unpaired two-tailed Mann-Whitney test using Stata Version 10.1. For experiments with multiple treatments (Figure 3BC, 5D, and S1B) ANOVA or Kruskal-Wallis (for data not normally distributed) with a Dunn's post-test yielded similar results as the indicated *t*-tests.

### Accession numbers

Proteins discussed in this manuscript are listed followed by their corresponding UniProtKB (Universal Protein Knowledgebase) number: MucA (MUCA_PSEAE), DinB (DPO4_PSEAE), AlgT/U (RPSH_PSEAE), and LexA (LEXA_PSEAE).

## Supporting Information

Figure S1 **Development of selection strategy to measure the frequency of mucoid conversion.** **A**, representative input non-mucoid and output mucoid PAO1*algD-cat* isolates following mucoid conversion assay. **B**, non-mucoid PAO1*algD-cat*, PAO1*algD-catΔdinB*, PAO1*algD-catΔmutS*, and PAO1*algD-catΔmutSΔdinB* were treated with sub-lethal hydrogen peroxide (H_2_O_2_, 0.1 µM) or Hank's Buffered Saline (HBSS) for one hour and the mucoid conversion frequency determined according to the *Materials and Methods*. All experiments were performed in triplicate on four independent occasions. Values are mean +/− SEM. Statistical analysis was carried out using an unpaired two-tailed student's *t*-test comparing the mucoid conversion frequencies of H_2_O_2_-treated PAO1*algD-cat* to HBSS-treated in **B** (* = p≤0.05, ** = *p*≤0.001).
(PNG)

Figure S2 **PMNs induce mucoid conversion independent of bacterial uptake and the oxidative burst response.** **A**, representative immunofluorescent images demonstrating cytochalasin D treated PMNs (stained with CytoTracker Blue) do not uptake PAO1*gfp* compared to DMSO treated PMNs (stained with CytoTracker Red). In **B** and **C,** phagocytosis of human peripheral PMNs was blocked by either treatment with cytochalasin D (**B**) or physical separation of PMNs from bacteria with transwells (**C**) and incubated with opsonized PAO1*algD-cat* (MOI 50). The kinetic oxidative burst response of PMNs was measured by luminol (relative luminescent units, RLU) over 60 minutes. All experiments were performed in triplicate on three independent occasions. Values are mean +/− SEM. For the statistical analysis of the kinetic oxidative burst response, the area under each curve was calculated ((RLU*min)^2^) for each treatment. Analysis was carried out using an unpaired two-tailed student's *t*-test to compare the oxidative burst response of either cytochalasin D compared to DMSO treated PMNs (**B**) or PMN + PAO1 together compared to separate (**C**). (* p≤0.05).
(TIFF)

Figure S3 **Immunoblot demonstrating detection of mature LL-37 in PMN Lysates.** PMN lysates were separated by SDS-PAGE on a 16.5% tris-tricine gel and mature LL-37 was detected by immunoblot analysis. Purified LL-37 (0.25 µM) was included as a positive control. Arrow indicates detection of mature LL-37 (4 KDa).
(TIFF)

Figure S4 **Electrophoretic mobility shift analysis of LL-37 interactions with** ***P. aeruginosa*** **DNA.** Increasing amounts of native LL-37 (top panel) and LL-37 variants (scrambled, middle panel and KR7-19E, bottom panel) were incubated with 5′ FAM labeled DNA fragments generated from the *mucA* gene (identical results were found with fragments generated from the *lecB* and *algU/T* genes (data not shown)). A representative image of three independent experiments and the position of free DNA and DNA bound by LL-37 are indicated.
(TIFF)

Movie S1 **Z-stack movie of confocal microscopy showing localization of LL-37 in** ***P. aeruginosa*** **cells.** Bacteria were treated +/− 0.25 µM LL-37, fixed, permeablized, stained for LL-37 (red) and visualized by 100× objective. Movie is a compilation of images taken sequentially through the Z-plane of the image every 0.16 µm.
(MOV)

Table S1 **Summary of** ***mucA*** **mutations induced by inflammatory factors.** Sequence analysis of the *mucA* gene from mucoid *P. aeruginosa* isolates derived from mucoid conversion assays.
(DOCX)

Table S2 **Summary of** ***mucA*** **mutations induced by LL-37.** Sequence analysis of the *mucA* gene from mucoid *P. aeruginosa* isolates treated with sub-lethal LL-37.
(DOCX)

Table S3 **qRT-PCR reveals sub-lethal LL-37 does not induce** ***P. aeruginosa*** **membrane or SOS stress responses.** Expression of *lexA*, *dinB*, and *algT* by non-mucoid PAO1 treated with sub-lethal LL-37 (0.25 and 1.25 µM) measured by qRT-PCR. Fold increase in relative copy number (RCN, compared to the housekeeping gene *rpsL*) from untreated cells is indicated. Values are the mean of at least three independent experiments performed in triplicate +/− SD. NA = not applicable. *Indicates a statistically significant difference in RCN compared to untreated cells using an unpaired student's *t*-test (*P*≤0.05).
(DOCX)

Table S4 **Bacterial strain table.** Bacteria and plasmids used in this study.
(DOCX)

Table S5 **Primer table.** Primers used in this study.
(DOCX)

Text S1 **Supporting Materials and Methods.** Description of materials and methods not described in the primary manuscript.
(DOCX)
