# Supplementary figures and images for: Cationic Antimicrobial Peptides Promote Microbial Mutagenesis and Pathoadaptation in Chronic Infections
Source: PLoS Pathog. 2014 Apr 24;10(4):e1004083. doi: 10.1371/journal.ppat.1004083 (PMC3999168; doi:10.1371/journal.ppat.1004083)

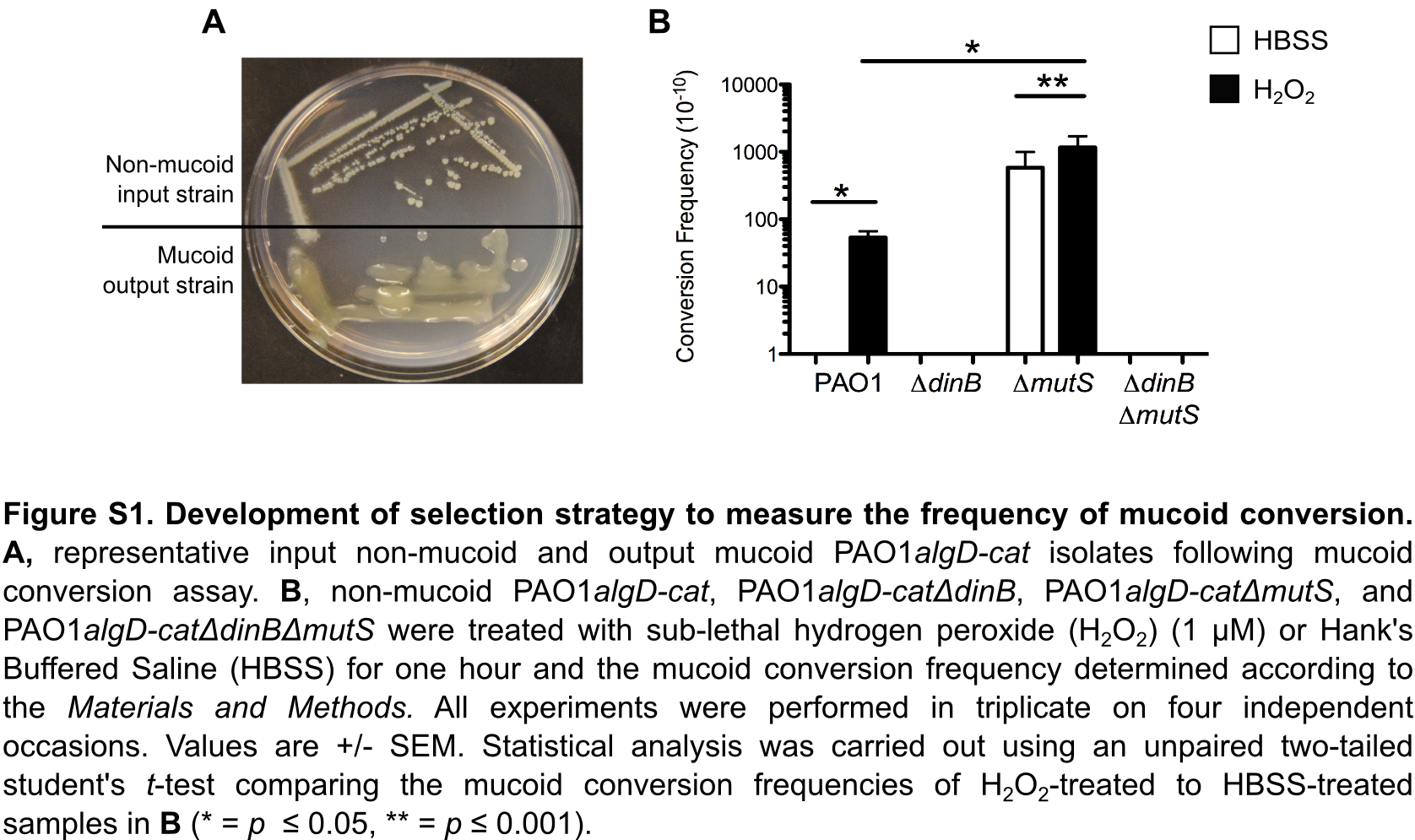

Supplement: Figure S1 — Development of selection strategy to measure the frequency of mucoid conversion. A, representative input non-mucoid and output mucoid PAO1algD-cat isolates following mucoid conversion assay. B, non-mucoid PAO1algD-cat, PAO1algD-catΔdinB, PAO1algD-catΔmutS, and PAO1algD-catΔmutSΔdinB were treated with sub-lethal hydrogen peroxide (H2O2, 0.1 µM) or Hank's Buffered Saline (HBSS) for one hour and the mucoid conversion frequency determined according to the Materials and Methods . All experiments were performed in triplicate on four independent occasions. Values are mean +/− SEM. Statistical analysis was carried out using an unpaired two-tailed student's t-test comparing the mucoid conversion frequencies of H2O2-treated PAO1algD-cat to HBSS-treated in B (* = p≤0.05, ** = p≤0.001). (PNG) [file ppat.1004083.s001.png]

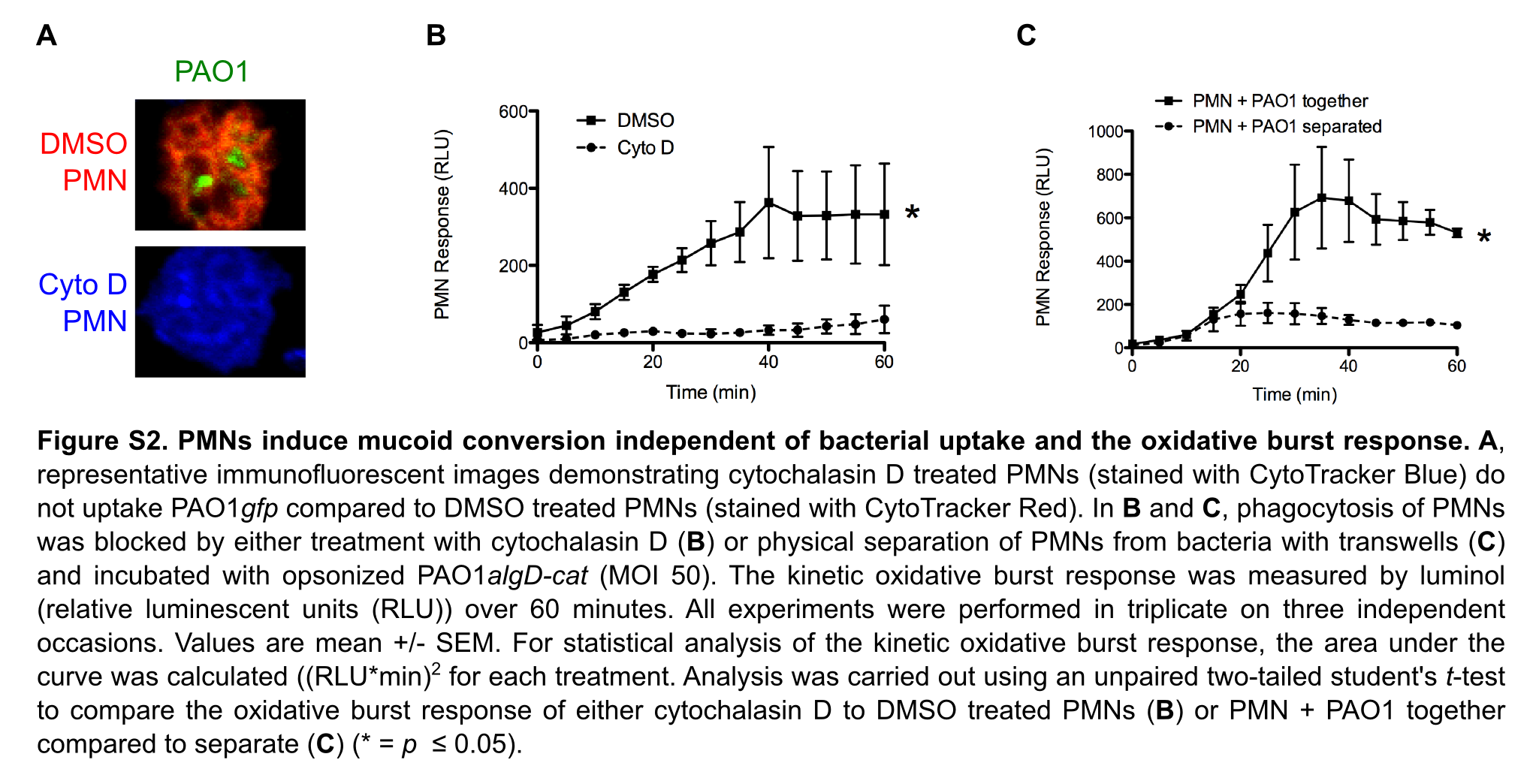

Supplement: Figure S2 — PMNs induce mucoid conversion independent of bacterial uptake and the oxidative burst response. A, representative immunofluorescent images demonstrating cytochalasin D treated PMNs (stained with CytoTracker Blue) do not uptake PAO1gfp compared to DMSO treated PMNs (stained with CytoTracker Red). In B and C, phagocytosis of human peripheral PMNs was blocked by either treatment with cytochalasin D (B) or physical separation of PMNs from bacteria with transwells (C) and incubated with opsonized PAO1algD-cat (MOI 50). The kinetic oxidative burst response of PMNs was measured by luminol (relative luminescent units, RLU) over 60 minutes. All experiments were performed in triplicate on three independent occasions. Values are mean +/− SEM. For the statistical analysis of the kinetic oxidative burst response, the area under each curve was calculated ((RLU*min)2) for each treatment. Analysis was carried out using an unpaired two-tailed student's t-test to compare the oxidative burst response of either cytochalasin D compared to DMSO treated PMNs (B) or PMN + PAO1 together compared to separate (C). (* p≤0.05). (TIFF) [file ppat.1004083.s002.tif]
