# Supplementary material for: Cationic Antimicrobial Peptides Promote Microbial Mutagenesis and Pathoadaptation in Chronic Infections
Source: PLoS Pathog. 2014 Apr 24;10(4):e1004083. doi: 10.1371/journal.ppat.1004083 (PMC3999168; doi:10.1371/journal.ppat.1004083)
Supplement: Table S1 — Summary of mucA mutations induced by inflammatory factors. Sequence analysis of the mucA gene from mucoid P. aeruginosa isolates derived from mucoid conversion assays. (DOCX) [file ppat.1004083.s006.docx]

| **Table S1. Summary of *mucA* mutations induced by inflammatory factors*.** | | | | | | |
| --- | --- | --- | --- | --- | --- | --- |
|  | Transitions | Transversions | Deletions | Insertions | WT | Total |
| H_2_O_2_ | 1 | 3 | 7 | 0 | 4 | 17 |
| O_2_• | 0 | 4 | 0 | 1 | 0 | 6 |
| PMN | 1 | 3 | 2 | 0 | 5 | 11 |
| PMN Lysate | 0 | 2 | 5 | 0 | 1 | 8 |
| Granules | 0 | 0 | 2 | 2 | 1 | 5 |
| *Changes compared to the *mucA* sequence of WT PAO1*algD-cat* parental strain. | | | | | | |
