# Supplementary material for: Cationic Antimicrobial Peptides Promote Microbial Mutagenesis and Pathoadaptation in Chronic Infections
Source: PLoS Pathog. 2014 Apr 24;10(4):e1004083. doi: 10.1371/journal.ppat.1004083 (PMC3999168; doi:10.1371/journal.ppat.1004083)
Supplement: Table S2 — Summary of mucA mutations induced by LL-37. Sequence analysis of the mucA gene from mucoid P. aeruginosa isolates treated with sub-lethal LL-37. (DOCX) [file ppat.1004083.s007.docx]

**Table S2. Summary of *mucA* mutations induced by LL-37.**

| **Strain** | ***mucA* mutation*** |
| --- | --- |
| **Frameshift** |  |
| LL-37 1.1 | ΔC at 184 |
| LL-27 2.1 | ΔT at 470 |
| LL-37 4.2 | ΔT at 546 |
| LL-37 6.1 | C insertion at 365 |
| LL-37 5.1 | Δ4 bp at 565 |
| LL-37 2.3 | 2 bp insertion at 529 |
| LL-37 7.1 | Δ5 bp at 225, ΔA at 231, Δ7 bp at 236 |
| **Other** |  |
| LL-37 1.2 | C→A at 531 |
| LL-37 4.1 | C→T at 31 |
| LL-37 6.2 | Δ17 bp at 229, Δ3 bp at 250, Δ3 at 256 |

*Changes compared to the *mucA* sequence of WT PAO1*algD-cat* parental strain.
