# Supplementary material for: Cationic Antimicrobial Peptides Promote Microbial Mutagenesis and Pathoadaptation in Chronic Infections
Source: PLoS Pathog. 2014 Apr 24;10(4):e1004083. doi: 10.1371/journal.ppat.1004083 (PMC3999168; doi:10.1371/journal.ppat.1004083)
Supplement: Table S3 — qRT-PCR reveals sub-lethal LL-37 does not induce P. aeruginosa membrane or SOS stress responses. Expression of lexA, dinB, and algT by non-mucoid PAO1 treated with sub-lethal LL-37 (0.25 and 1.25 µM) measured by qRT-PCR. Fold increase in relative copy number (RCN, compared to the housekeeping gene rpsL) from untreated cells is indicated. Values are the mean of at least three independent experiments performed in triplicate +/− SD. NA = not applicable. *Indicates a statistically significant difference in RCN compared to untreated cells using an unpaired student's t-test (P≤0.05). (DOCX) [file ppat.1004083.s008.docx]

| **Table S3. qRT-PCR reveals sub-lethal LL-37 does not induce *P. aeruginosa* membrane or SOS stress responses^†^.** | | | | |
| --- | --- | --- | --- | --- |
|  | **0.25 μM LL37** | **1.25 μM LL37** | **Mitomycin C** | **D- cycloserine** |
| ***lexA*** | 1.3 +/- 0.7 | 13.7 +/- 23.2 | 307.0 +/- 50* | NA |
| ***dinB*** | 0.8 +/- 0.2 | 1.0 +/- 1.4 | 6.0 +/- 1.7* | NA |
| ***algT*** | 1.4 +/- 0.1 | 0.5 +/- 0.5 | NA | 2.5+/-1.1* |
| †Fold increase in relative copy number (RCN, compared to the housekeeping gene *rpsL*) from untreated cells. Values are the mean of at least three independent experiments performed in triplicate +/- SD. NA = not applicable. *Indicates a statistically significant difference in RCN compared to untreated cells using an unpaired student’s *t*-test (*P* ≤ 0.05). | | | | |
