# Supplementary material for: Cationic Antimicrobial Peptides Promote Microbial Mutagenesis and Pathoadaptation in Chronic Infections
Source: PLoS Pathog. 2014 Apr 24;10(4):e1004083. doi: 10.1371/journal.ppat.1004083 (PMC3999168; doi:10.1371/journal.ppat.1004083)
Supplement: Table S4 — Bacterial strain table. Bacteria and plasmids used in this study. (DOCX) [file ppat.1004083.s009.docx]

| **Table S4. Bacterial strains and plasmids used in this study^*^** | | |
| --- | --- | --- |
| **Strain or plasmid** | **Phenotype, genotype and/or description** | **Source** |
| *P. aeruginosa* strains |  |  |
| PAO1 Cm^S^ | Alg-, prototroph, Cm^S^ | [7] |
| WFPA934 | Alg-, PAO1 Cm^S^, *algD-cat* | This study |
| WFPA936 | Alg-, PAO1 Cm^S^, *algD-cat,* ∆*mutS* | This study |
| WFPA937 | Alg-, PAO1 Cm^S^, *algD-cat,* ∆*dinB*::*aacC1* | This study |
| WFPA938 | Alg-, PAO1 Cm^S^, *algD-cat,* ∆*mutS* ∆*dinB*::*aacC1* | This study |
| PDO300 | Alg+, PAO1∆*mucA22* | [15] |
| FRD1 | Alg+, FRD1 ∆*mucA22,* clinical isolate | [16] |
| FRD440 | Alg-, FRD1 ∆*mucA22, algT::Tn501* | [17] |
|  |  |  |
| *E. coli* strains |  |  |
| UTI89 | prototypic cystitis uropathogenic *E. coli* isolate | [18] |
| SM10/λpir | *thi recA thr leu tonA lacY supE* RP4–2-Tc::Mu1::*pir* Km^R^ | Laboratory strain |
|  |  |  |
| Plasmids |  |  |
| pKK61 | pCP19 (*oriV*[RK2], TcR) P*algD-cat* | [19] |
| pHL16 | Ap^R^, Gen^R^; pHL13 bearing *P. aeruginosa ΔdinB::aacC1* (Gen^R^) | [1] |
| pEX18Ap | Ap^R^ (Carb^R^); suicide replacement vector containing *B. subtilis sacB* gene | [20] |
| pHL170 | Ap^R^ (Carb^R^); pEXAp bearing *P. aeruginosa* in-frame deletion of *mutS* | This study |
| pHERD20T | pUCP20T P*lac* replaced with 1.3-kb AflII-EcoRI fragment of *araC*-PBAD cassette (5,087 bp) | [21] |
| pHERD20T-*mucA* | *mucA* in pHERD20T EcoRI/HindIII | [21] |
| ^*^Alg-, non-mucoid phenotype, Alg+, mucoid phenotype | | |
