# Supplementary material for: Cationic Antimicrobial Peptides Promote Microbial Mutagenesis and Pathoadaptation in Chronic Infections
Source: PLoS Pathog. 2014 Apr 24;10(4):e1004083. doi: 10.1371/journal.ppat.1004083 (PMC3999168; doi:10.1371/journal.ppat.1004083)
Supplement: Table S5 — Primer table. Primers used in this study. (DOCX) [file ppat.1004083.s010.docx]

| **Tables S5. Primers used in this study** | | |
| --- | --- | --- |
| **Primer** | **Sequence (5’-3’)** | **Source** |
| *mucA*upF | TGTTGCGGGATGAGATCGAGG | This study |
| *mucA*dnR | GGGTGGAGAAGCTGCCATTG | This study |
| *mucA*1F21 | GGATCTTCCGCGCTCGTGAAG | This study |
| r*psL*-RT - F1 | ACCACGCCGAAAAAGCCGAA | This study |
| *rpsL*-RT - R1 | CCGCCACGGATCAGCACTAC | This study |
| *algT*-RT - F1 | GGACATCGCCCTCGGCAT | This study |
| *algT*-RT - R1 | CGACGAAGGCACAGGTGGC | This study |
| *dinB*-RT - F1 | CGACTGTTTCTAGCCGCCCTC | This study |
| *dinB*-RT - R1 | CGCCTCGTGCTGCAAGTGG | This study |
| *lexA*-RT - F1 | CTCCTTCATCAAGCGCTGCCTGG | This study |
| *lexA*RT - R1 | GTTCGGCGACTTGAAGCCGAGTT | This study |
