# Supplementary material for: Cationic Antimicrobial Peptides Promote Microbial Mutagenesis and Pathoadaptation in Chronic Infections
Source: PLoS Pathog. 2014 Apr 24;10(4):e1004083. doi: 10.1371/journal.ppat.1004083 (PMC3999168; doi:10.1371/journal.ppat.1004083)
Supplement: Text S1 — Supporting Materials and Methods. Description of materials and methods not described in the primary manuscript. (DOCX) [file ppat.1004083.s011.docx]

**Supporting Materials and Methods**

**Bacterial Strains, Plasmids, and Growth Conditions.** The bacterial strains used in this study are described in the Table S4. *Pseudomonas aeruginosa* strains were routinely grown in M63 media [1,2] and *Pseudomonas* Isolation Agar (PIA) (Difco) was utilized for growth on solid medium. *Escherichia coli* was grown in Luria-Bertani (LB). The following antibiotics were used at the indicated concentrations with *E. coli* strains: ampicillin and gentamycin at 100 μg/ml and rifampin at 100 μg/ml. For *P. aeruginosa* strains: gentamycin was used at 100 μg/ml, carbenicillin at 300 μg/ml, chloramphenicol at 250 μg/ml and rifampin at 100 μg/ml. Reagents were purchased from Sigma-Aldrich, St. Louis, MO, unless otherwise indicated.

**Genetic Techniques.** DNA extraction, treatment with modifying enzymes and restriction endonucleases, ligation with T4 ligase, and transformation of *E. coli* were performed using standard methods unless otherwise stated. PAO1*algD-cat* was constructed by integrating a construct containing the *algD* promoter fused to a chloramphenicol acetyl-transferase cassette (pCP19 (*oriV*[RK2], TcR) [3-5] into the neutral *attB* site of the wild-type chloramphenicol sensitive variant of PAO1 [6,7] and mutant *P. aeruginosa* strains: PAO1*algD-cat* ∆*mutS*, ∆*dinB*::*aacC1*, and ∆*mutS*∆*dinB*::*aacC1*. PAO1 ∆*mutS*, ∆*dinB*, and ∆*mutS*∆*dinB* were generated using standard mating and sucrose selection gene replacement strategies of the respective wild-type genes with ∆*dinB*::*aacC1* from pHL16 [1,8] and ∆*mutS* with pHL170. pHL170 was constructed by using an unmarked, nonpolar deletion strategy described previously [3,9]. Flanking regions of *mutS* were amplified using primers *mutS* F and *mutS* R. The resultant PCR product was ligated into the suicide vector, pEX18Ap, via *Hin*dIII restriction site. The plasmid pHL170 was verified by sequence analysis.

**PCR and sequencing.** Genomic DNA was harvested from the mucoid variants isolated in the mucoid conversion assay using the Wizard genomic purification kit (Promega). PCR amplification was performed using *mucA-*specific primers *mucA*upF and *mucA*dnR. After verification of PCR product by agarose gel electrophoresis, the PCR products were sequenced by The Ohio State University Medical Center Nucleic Acid Core using Sanger sequencing techniques with *mucA*upF, *mucA*dnR, *mucA*1F21. The sequence data produced for mucoid variants were then aligned with *mucA* gene sequence of the non-mucoid PAO1*algD-cat* parental to determine if and/or where mutations occurred in the *mucA* gene of the variants.

**PMN studies.** Human PMNs and serum were obtained from healthy adult donors using an approved IRB protocol (2009H0314) at The Ohio State University and isolated according to previously described protocols [6,10]. Promyelocytic HL-60 cells were acquired from the American Type Culture Collection (ATCC)(CCL-240), and maintained according to ATCC instructions. Cells were terminally differentiated into PMNs by the addition of 1.25% dimethyl sulfoxide (DMSO) for five days [8,11]. All PMNs were resuspended in HBSS, counted on a haemocytometer and seeded (2 x10^5^) on a 96-well microtitre plate for the luminol chemiluminescence assay and on a 6-well microtitre plate for the mucoid conversion assay. Mid-log phase *P. aeruginosa* suspensions were opsonized with 10% fresh human autologous serum at 37°C for 30 min, added (MOI of 50) to the wells and centrifuged at 100 *g* for 2 min at 4°C to synchronize the infection. The mucoid conversion frequency was determined by incubation of PMNs and bacteria for one hour at 37°C, 5% CO_2_ and the whole culture was then plated as described in the *Materials and Methods*. The luminol chemiluminescence assay was used to detect intracellular and extracellular ROS generated by neutrophils upon interaction with *P. aeruginosa* by incubation in the presence of 50 mM of luminol [9,11]. The relative amount of ROS generated by neutrophils was detected at regular intervals over 60 min by measuring the luminescence. Relative light units (RLU) were plotted as a function of time to evaluate chemiluminescence rate [10]. Chemical inhibition of phagocytosis was accomplished by incubation with cytochalasin D (5 μM) or DMSO as a carrier control with PMNs for 30 minutes prior to infection with *P. aeruginosa* strains [11]. Inhibition of phagocytosis was confirmed by visualization of bacteria-phagocyte association by confocal microscopy (Olympus FV 1000) using a 60x oil objective. Cytochalasin D-treated neutrophils were stained with CytoTracker Blue and DMSO-treated neutrophils were stained with CytoTracker Red and infected with GFP-tagged PAO1 as described above. Physical separation with transwells was achieved by placing a Corning® Transwell® polycarbonate membrane insert (0.4 μm) close to the bottom of the well, preventing direct contact of the bacteria with the PMNs seeded below [11]. The oxidative burst response was blocked by the NADPH oxidase inhibitor, diphenyleneiodonium chloride (DPI) by incubation of PMNs for 30 min with 10 μM DPI prior to infection [2,12]. To obtain PMN lysates, 2.5 x 10^8^ PMNs were resuspended in HBSS followed by physical disruption at 4°C and centrifugation at 100 *g* for 10 minutes at 4°C to remove unlysed cells. To isolate soluble granule components, granules were then pelleted by centrifugation at 30,000 *g* for 30 minutes at 4°C and resuspended in HBSS. On ice, granules were periodically sonicated for 30 seconds followed by lysis with ZIROX-70 Zirconium Silicate Beads (0.1 mm) by vortexing vigorously for four minutes followed by centrifugation at 30,000 *g* for 30 minutes at 4°C [4,5,13]. The supernatant with released granule components was then removed and granule isolation and lysis confirmed by assaying for the presence of myeloperoxidase (MPO) with an MPO fluorometric detection kit according to the manufacturers’ instructions (Enzo Life Sciences) and the luminol assay (see above) was performed to confirm no ROS were present.

**Immunoblot for LL-37.** PMN lysate samples were separated on an SDS-PAGE gel (16.5% Ready Gel Tris-Tricine Precast Gels, Bio-Rad, Hercules, CA). Samples were transferred to a PVDF membrane (Bio-Rad, Hercules, CA) and blocked in 3% skim milk. Membranes were incubated with mouse anti-LL-37 (Santa Cruz Biotechnology, Dallas, TX) overnight at 4°C, washed, and incubated with goat anti-mouse IgG (H+L) HRP conjugate (Santa Cruz Biotechnology, Dallas, TX). Membranes were washed and peroxidase activity was detected using SuperSignal West Femto Chemiluminescent Substrate (Thermo Scientific, Rockford, IL).

**RNA harvest and qRT-PCR**. PAO1 was grown to mid-log phase and 1 x 10^6^ cells were incubated with sub-lethal LL-37 (0.25 and 1.25 μM), mitomycin C (1 μg/ml), 400 μg/ml D-cycloserine or 10 mM sodium phosphate buffer (pH 6.2)(SPB) for one hour, washed and total RNA was harvested using Qiagen RNeasy® Mini Columns (Qiagen) according to the manufacturer’s instructions. Following purity confirmation by gel electrophoresis, 1 μg of this RNA was used as a template to prepare cDNA using SuperScript® III First-Strand Synthesis SuperMix (Invitrogen). Real-time PCR was carried out using SYBR Green (Invitrogen) with primers: *algT*-RT - F1, *algT*-RT – R1, *dinB*-RT – F1, *dinB*-RT4 – R1, *lexA*-RT – F1 and  *lexA*-RT2 – R1 (Table S5). Reactions were performed and analyzed using BioRad CFX96 Real Time System. For each RNA sample, mRNA levels were normalized against *rpsL* mRNA levels, which were determined using primers r*psL*-RT - F1, *rpsL*-RT - R1. Once normalized against *rpsL*, data were presented as a fold-increase over SPB treated.

**Electrophoretic mobility shift assay.** PCR amplification with 5’-6-carboxyfluorescein (6-FAM)-labeled DNA primers amplified regions of *mucA*, *lecB* and *algT* to generate fluorescein-labeled PCR products. Electrophoretic mobility shift assays (EMSA) were performed as previously published [7,14]. In brief, 5 ng of protein was combined with 150 fmol of DNA fragment in EMSA binding buffer (20 mM Tris-HCl [pH 8.0], 200 mM NaCl, 20 mM MgCl_2_, 20% glycerol) and 750 ng poly(dI-dC) and allowed to bind for 10 min at room temperature. The samples were resolved on a 4% polyacrylamide native gel and imaged on a Typhoon fluorescent scanner with 488-nm excitation and high sensitivity. Densitometry was performed using ImageJ and the K_d_ (dissociation constant) was calculated based on the equation generated by the best-fit curve using a single-ligand binding model.

**Supporting References**

1. Sanders LH, Rockel A, Lu H, Wozniak DJ, Sutton MD (2006) Role of *Pseudomonas aeruginosa* *dinB*-encoded DNA polymerase IV in mutagenesis. J Bacteriol 188: 8573–8585. doi:10.1128/JB.01481-06.

2. O'Toole GA, Kolter R (1998) Initiation of biofilm formation in *Pseudomonas fluorescens* WCS365 proceeds via multiple, convergent signalling pathways: a genetic analysis. Mol Microbiol 28: 449–461.

3. Hoang T, Karkhoff-Schweizer R, Kutchma A, Schweizer H (1998) A broad-host-range Flp-FRT recombination system for site-specific excision of chromosomally-located DNA sequences: applications for isolation of unmarked *Pseudomonas aeruginosa* mutants. Gene 212: 77–86.

4. Wozniak D, Ohman D (1991) *Pseudomonas aeruginosa* AlgB, a two-component response regulator of the NtrC-family, is required for algD transcription. J Bacteriol 173: 1406–1413.

5. Wood LF, Leech AJ, Ohman DE (2006) Cell wall-inhibitory antibiotics activate the alginate biosynthesis operon in *Pseudomonas aeruginosa*: Roles of sigma (AlgT) and the AlgW and Prc proteases. Mol Microbiol 62: 412–426. doi:10.1111/j.1365-2958.2006.05390.x.

6. Mishra M, Byrd MS, Sergeant S, Azad AK, Parsek MR, et al. (2012) *Pseudomonas aeruginosa* Psl polysaccharide reduces neutrophil phagocytosis and the oxidative response by limiting complement-mediated opsonization. Cell Microbiol 14: 95–106. doi:10.1111/j.1462-5822.2011.01704.x.

7. Holloway BW, Krishnapillai V, Morgan AF (1979) Chromosomal genetics of Pseudomonas. Microbiol Rev 43: 73.

8. Collins SJ, Ruscetti FW, Gallagher RE, Gallo RC (1978) Terminal differentiation of human promyelocytic leukemia cells induced by dimethyl sulfoxide and other polar compounds. Proc Natl Acad Sci U S A 75: 2458.

9. Engels W, Endert J, Kamps MAF, Van Boven CPA (1985) Role of lipopolysaccharide in opsonization and phagocytosis of *Pseudomonas aeruginosa*. Infection and Immunity 49: 182.

10. Mohapatra NP, Soni S, Rajaram MVS, Dang PMC, Reilly TJ, et al. (2010) Francisella Acid Phosphatases Inactivate the NADPH Oxidase in Human Phagocytes. J Immunol 184: 5141–5150. doi:10.4049/jimmunol.0903413.

11. Lindmark H, Johansson KC, Stöven S, Hultmark D, Engström Y, et al. (2001) Enteric bacteria counteract lipopolysaccharide induction of antimicrobial peptide genes. J Immunol 167: 6920–6923.

12. Porro GA, Lee J-H, de Azavedo J, Crandall I, Whitehead T, et al. (2001) Direct and indirect bacterial killing functions of neutrophil defensins in lung explants. Am J Physiol Lung Cell Mol Physiol 281: L1240–L1247.

13. Lominadze G (2005) Proteomic Analysis of Human Neutrophil Granules. Molecular & Cellular Proteomics 4: 1503–1521. doi:10.1074/mcp.M500143-MCP200.

14. Waligora EA, Ramsey DM, Pryor EE, Lu H, Hollis T, et al. (2010) AmrZ beta-sheet residues are essential for DNA binding and transcriptional control of *Pseudomonas aeruginosa* virulence genes. J Bacteriol 192: 5390–5401. doi:10.1128/JB.00711-10.

15. Mathee K, Ciofu O, Sternberg C, Lindum PW, Campbell JI, et al. (1999) Mucoid conversion of *Pseudomonas aeruginosa* by hydrogen peroxide: a mechanism for virulence activation in the cystic fibrosis lung. Microbiology 145 : 1349–1357.

16. Ohman D, Chakrabarty A (1981) Genetic mapping of chromosomal determinants for the production of the exopolysaccharide alginate in a *Pseudomonas aeruginosa* cystic fibrosis isolate. Infect Immun 33: 142–148.

17. Flynn J, Ohman D (1988) Cloning of genes from mucoid *Pseudomonas aeruginosa* which control spontaneous conversion to the alginate production phenotype. J Bacteriol 170: 1452–1460.

18. Mulvey MA, Schilling JD, Hultgren SJ (2001) Establishment of a persistent *Escherichia coli* reservoir during the acute phase of a bladder infection. Infect Immun 69: 4572–4579. doi:10.1128/IAI.69.7.4572-4579.2001.

19. Wozniak DJ, Ohman DE (1991) *Pseudomonas aeruginosa* AlgB, a two-component response regulator of the NtrC family, is required for algD transcription. J Bacteriol 173: 1406–1413.

20. Hoang TT, Karkhoff-Schweizer RR, Kutchma AJ, Schweizer HP (1998) A broad-host-range Flp-FRT recombination system for site-specific excision of chromosomally-located DNA sequences: application for isolation of unmarked *Pseudomonas aeruginosa* mutants. Gene 212: 77–86.

21. Qiu D, Damron FH, Mima T, Schweizer HP, Yu HD (2008) PBAD-Based Shuttle Vectors for Functional Analysis of Toxic and Highly Regulated Genes in *Pseudomonas* and *Burkholderia spp*. and Other Bacteria. Appl Environ Microbiol 74: 7422–7426. doi:10.1128/AEM.01369-08.
